# Nerve Removal versus Conservative Treatment

**Published:** 1906-02

**Authors:** R. B. Harris

**Affiliations:** Cartersville, Ga.


### NERVE REMOVAL VERSUS CONSERVATIVE TREATMENT.

By R. B. Harris, B. S., D. D. S., Cartersville, Ga.

(Georgia Dental Society Paper.)

In presenting this subject to you, I desire to so define conservative treatment as that the limitations will be understood to apply to diseased conditions of the pulp and to all cases of actual exposure of pulp tissue.

It is not my purpose to preesnt any new theories in dealing with these conditions, for it does seem to me, that before now, we have had enough and to spare of bursting bubbles on a turbid stream.

Conservatism, in many phases of life, plays a delusive hand, and with mask hidden face is often a synonym of no accountism or deception. In some of the conservative treatment of pulps, it is probable that one or the other of the last named conditions largely obtains.

When the very conservative dentist invades that forbidden domicile of the pulp, he gets ready his very conservative remedies of pastes and cavity stoppers and caps, and he says, "Little nerve, I beg your pardon, I did not intend to uncover your virgin form and expose your body to the rude gaze of an unfriendly world; but I have intruded a little just at one corner of your home. Be very quiet now while I repair this hole in the roof of your bed chamber, and don't raise any disturbance to trouble the tired dentist, for you will feel better after a while. Soon Mother Nature, in her solicitude and anxiety for your better protection, will, with loving hand and matronly care, begin to weave about you a barrier of redeposited dentine, and the walls of your home will again be garnished and adorned by the hand of a master builder."

Under this deception, and quieted for the time by some soothing lotion, the wounded nerve may cease to cry out and patiently wait for the fulfillment of the promises made. Ere the deception is realized it is in the throes of death. Sometimes its agonies are beggarly of description and for relief it must undergo that awful procedure of puncturing, with days and weeks of sickness and suffering to follow, before the welcome end of its life can release it from its woes. But often its trust in the conservative man is so implicit that it refuses to complain until the vital spark is extinct, and its once happy home becomes its grave. There it may slumber for days, weeks or months, until Nature rebels at the treatment of her child and casts forth its putrid remains in the office and nostrils, oftentimes of an innocent man, but fails not to write condemnation above, beneath and around the name of the evil doer.

Or if the encroachment made by the conservative dentist is too great to admit any hope of possible recovery from the damage, he gets ready his very conservative embalming materials and he says, "Poor little nerve! Your injuries have been so great you can never recover the happy life you have lived in this beautiful chamber of ivory and pearl; but it would be too bad, with ruthless hand, to take you away from this charming knew and loved you not. So be quiet now and I will lull you abode, and put in your home an alien and a stranger who gently to rest with cocaine so that you will not suffer a pang, and then I will embalm your body with sweet scented savours like unto iodoform and thymol, so that you shall remain forever in perfect preservation in your silent and peaceful home. And I will place at the door of your chamber an antiseptic, always to stand sentinel with drawn and glittering sword to protect you from any ghouls that might seek to spy out or prey upon your remains.

In the past, prophets have arisen to tell us how, that after much time and study spent in their chemical laboratories, and after months and years of scrupulously kept data and carefully observed results, they have succeeded at last, and have wrought out a formula to make life's walk easy to the dentist and save the patient from agonies untold. In our eagerness to walk the easy way as well as to save our patients from an operation that was formerly sometimes unpleasant, some of us have listened with enchanted ear to the tales we had been told, and cautiously ventured to handle nerves the easy way. In rare cases where thorough removal is impossible we may employ these methods, but the only ground we can justly assume for so doing is that it gives us the second chance to save the tooth, and rest assured that some day, soon or late, the second chance will demand the attention of some other dentist. Capped nerves may live for some men, but their devotion to me is greater—they die for me.

Leaving the question of nerve salvation, I will briefly allude to our real mission—tooth salvation. Whatever ground we may have formerly had for deferring nerve removal and temporizing with so many things to avoid it, there is at least now little room for so doing. When at all in doubt as to the absolute safety and future comfort of the pulp, make the operation of removal. Make it confidently, make it fearlessly, make it boldly. The manner of the operator has much to do with a successful operation. A deferred, debated, hesitating operation is a half failure to begin with, for the patient becomes nervous and restive under it.

At this day why they should look upon nerve removal with dread, I am at a loss to know, nevertheless, they do. For that reason I have ceased to tell a patient that I am going to remove a nerve. When I find it necessary to do so, I make the removal and tell them afterwards. Try it, and it will delight you to practice such pleasant surprises. If called upon to give the method I would, without material change, give the one submitted by Dr. Barker in his most admirable paper last year in Athens.

